# Characterization of an Electrode-Type Tactile Display Using Electrical and Electrostatic Friction Stimuli

**DOI:** 10.3390/mi12030313

**Published:** 2021-03-17

**Authors:** Seiya Komurasaki, Hiroyuki Kajimoto, Fusao Shimokawa, Hiroki Ishizuka

**Affiliations:** 1 Division of Intelligent Mechanical Systems Engineering, Graduate School of Engineering, Kagawa University, 2217-20 Hayashi-cho, Takamatsu, Kagawa 761-0396, Japan; s19g511@stu.kagawa-u.ac.jp; 2 Department of Human Communication, The University of Electro-Communications, 1-5-1 Chofugaoka, Chofu, Tokyo 182-8585, Japan; kajimoto@kaji-lab.jp; 3 Department of Engineering and Design, Kagawa University, 2217-20 Hayashi-cho, Takamatsu, Kagawa 761-0396, Japan; shimokawa.fusao@kagawa-u.ac.jp; 4 Department of Mechanical Science and Bioengineering, Division of Bioengineering, Graduate School of Engineering Science, Osaka University, 1-3 Machikaneyama, Toyonaka, Osaka 560-8531, Japan

**Keywords:** tactile display, electrical stimulus, electrostatic friction stimulus, multiple stimuli, electrode-type

## Abstract

Unlike tactile displays that use mechanical actuators, electrode-type tactile displays can be easily integrated and miniaturized because they consist of electrodes and insulators. Electrical tactile displays only require electrodes and use an electric current to stimulate vibration or pressure. Likewise, electrostatic friction tactile displays also only require electrodes and an insulator and can induce changes in friction between the display and a fingerpad. We have developed a tactile display that integrates electrical and electrostatic friction stimulation owing to their affinity to microfabrication techniques. This tactile display can provide both pressure and friction at the same time. In this study, we presented an elongated bar shape via the tactile display to experimental participants. The experimental results showed that a tactile display employing multiple stimuli as opposed to a single stimulus can induce the perception of larger shapes.

## 1. Introduction

Haptic and tactile technologies have attracted the interest of both researchers and industry because these technologies have been considered to be next-generation information technologies. One such technology, tactile displays, which provide tactile information to users, have been studied for several decades [1,2,3]. As practical applications that induce simple vibratory feedback, tactile displays can improve the quality of audio–visual content, such as video games and movies, as well as the operability of information devices, such as smart phones and tablet devices. In the future, tactile displays are expected to have more advanced applications, such as in teleoperations, for which such displays can enable the transmission of tactile information between remote areas [4,5]. Current tactile displays, however, are only capable of transmitting simple tactile senses, such as vibration, friction, and temperature, according to their principles.

Vibratory tactile displays provide a vibratory stimulus, with the displacement change of mechanical actuators or current stimulus to mechanoreceptors inside the skin. This vibratory stimulus can provide not only simple vibratory feedback but also surface texturing [6]. For example, Jang et al. arrayed Piezo actuators on a smartphone to enhance mobile tactile interaction [7]. Similarly, Lévesque et al. developed a tactile display consisting of an array of Piezo actuators. This tactile display was capable of conveying lateral vibrations to users, inducing lateral skin deformations [8]. Lévesque and colleagues further evaluated the performance of their tactile display as a Braille display. Zhao et al. developed a tactile display consisting of a range of small-shape memory alloy actuators [9]. Later, one of the authors of the present work developed a tactile display using nichrome wires to induce thermal expansion [10]. This tactile display had the capacity to provide a vibratory stimulus of up to 320 Hz. Several research groups have focused on electrostatic force actuators as they require only two thin electrodes and an insulator to produce a high-frequency response. Shao et al., for instance, combined electrostatic force actuators and a liquid-based displacement amplification mechanism to generate a tactile stimulus over a large area and with great force [11]. Tomita et al. fabricated a tactile display with an electrostatic force actuator consisting of conductive strings and a thin plastic film [12]. Although the displacement caused by this electrostatic actuator was small, it was nonetheless sufficient to stimulate a fingertip, which is a relatively sensitive part of the human body. Sonar et al. applied a pneumatic actuator to a tactile display to achieve large displacement, although this actuator responded more slowly than the other actuators mentioned above [13].

The primary shortcoming of tactile displays that incorporate mechanical actuators is their bulkiness. Although the size of a single tactile stimulator is small, tactile displays with mechanical actuators can be as large as a few cm^3^ [7,8,11], and while the tactile display that incorporates a pneumatic actuator is amenable to microfabrication and can be easily miniaturized [13], it requires a large air compressor and pneumatic valves. Thus, this tactile display is also too large. Consequently, these tactile displays, when applied to stand-alone use, can only provide a tactile sense or be used in virtual and smart gloves. Considering the applications of devices used in daily life, such as smartphones and tablet devices, tactile displays that are small enough to be integrated into these devices are greatly needed.

Unlike tactile displays that rely on mechanical actuators, tactile displays incorporating an electrical stimulus have the advantage of miniaturization because they only require electrodes. These displays can be made from microfabrication technologies and transparent materials, such as indium tin oxide, for use in the touchscreens of information devices. Additionally, as they can still provide the requisite sense of vibration or pressure [14,15], electrical stimulus-based tactile displays can replace those that rely on mechanical actuators. That said, the stability of the electrical stimulus must be improved [16]. Typically, these tactile displays require at least two electrodes: one connected to a high-voltage terminal, and one that is grounded. By applying electric current to the contacted skin, these electrodes can stimulate mechanoreceptors inside the skin, which perceive tactile senses, thereby conveying such tactile senses to a user.

Friction tactile displays, on the other hand, modulate the frictional force between the tactile display and the fingertip via the squeeze film effect or electrostatic friction. Tactile displays that use the squeeze film effect reduce the frictional force on their surface with high-frequency vibration plates. Biet et al. revealed that a tactile plate can be used to reproduce a programmable tactile sense, and additionally demonstrated that slippery surfaces could be successfully reproduced using the tactile plate [17]. Tactile displays that rely on electrostatic friction consist of an insulator layer and an electrode, the latter of which generates an electrostatic force when in contact with a fingertip, thereby increasing the resultant frictional force between the tactile display and the fingertip [18,19]. As opposed to the tactile displays that use the squeeze film effect, those that rely on an electrostatic force have a simpler structure because they only require electrodes covered with an insulator and can thus be incorporated into the touchscreens of information devices, much like electrical stimulus-based tactile displays.

In summary, tactile displays that rely on an electrical stimulus or electrostatic friction can be applied to information devices, but the tactile senses they convey, such as pressure and friction, are simple. It is known that the tactile perception of surfaces is determined by a combination of tactile stimuli, such as vibration, friction, and temperature [20,21]. Therefore, in order to reproduce more realistic tactile senses, each tactile stimulus must be independently controlled and provided. Considering that roughness and friction are independently perceived, and that surface textures are determined by roughness and friction [22,23,24,25,26,27], we have focused on the presentation of vibration, which is related to roughness, and on the presentation of friction.

**Figure 1:**
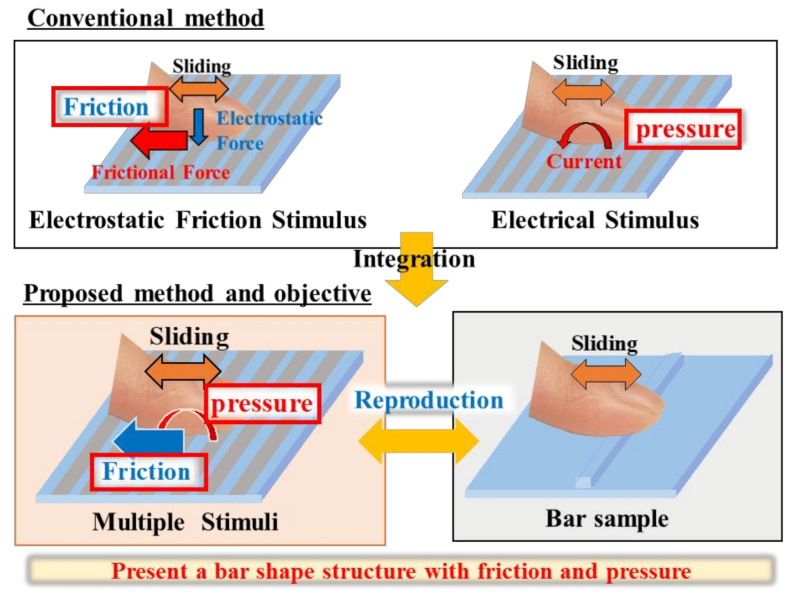
The concept and objective of the study.

In a previous study, we developed an integrated tactile display using electrical and electrostatic friction stimuli, considering their affinity to information devices and multiple tactile stimuli [28], as shown in the bottom-left panel of Figure 1. According to related studies, electrical and electrostatic friction stimuli can provide the sense of pressure and friction, respectively, as shown at the top of Figure 1. Our tactile display can provide both an electrical stimulus and an electrostatic friction stimulus while controlling the roughness reproduced by the electrical stimulus and the electrostatic friction stimulus independently. Ito et al. proposed a similar integrated tactile display using vibrations generated by a mechanical actuator and the friction generated by an electrostatic force [27]. However, their tactile display was bulky, and its stimulus resolution was poor. On the other hand, owing to the principles of electrical and electrostatic stimuli, our tactile display requires only electrodes for both stimuli. Thus, via the microfabrication technique, these stimulators can be miniaturized and can attain a higher resolution. Although we outlined the fundamental characteristics of this tactile display in our previous study, the tactile sense it provides has yet to be investigated. One of the authors of the present work earlier revealed that a single electrical stimulus could reproduce bar shapes [29]. Our proposed tactile display not only provides this electrical stimulus to reproduce bar shapes but can also enhance bar shapes with the presentation of friction, as shown in the bottom-right panel of Figure 1. In contrast, in other studies in which surfaces were reproduced with an electrostatic friction stimulus, only fine surfaces were sufficiently reproduced [30,31]. Additionally, it was found that the electrostatic friction stimulus was unsuitable for the presentation of three-dimensional shapes.

In the current study, we first determined the conditions needed for both an electrical stimulus and an electrostatic friction stimulus to provide pressure and friction. We then asked subjects to compare the provided multiple tactile stimuli with real bar shapes in order to determine the reproduction capability of the proposed tactile display, which has not yet been investigated.

## 2. Design and Principle

**Figure 2:**
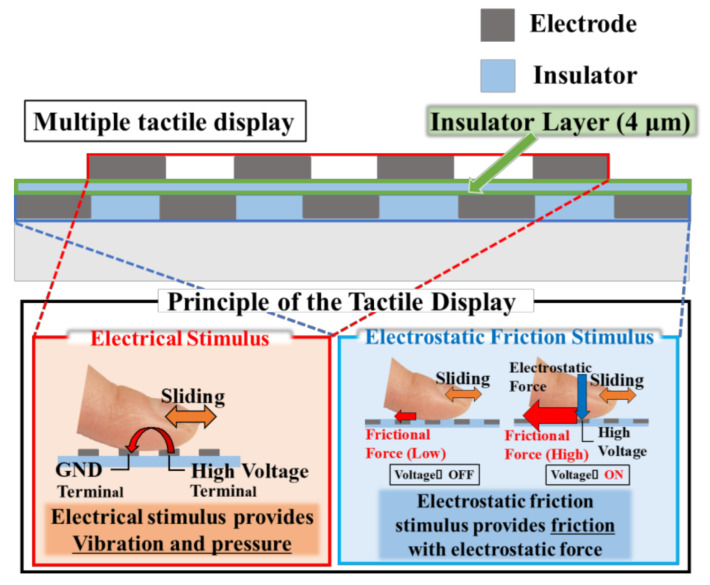
The principle of the proposed multiple tactile display.

Figure 2 depicts the structure and principle of the proposed tactile display. The electrodes used to stimulate electrical and electrostatic friction are alternately arranged, and both can simultaneously contact the skin when users slide their fingerpad on the tactile display, as shown at the bottom of Figure 2. In the following section, we explain the principles of the electrical and electrostatic friction stimuli.

On the bottom of the tactile display, electrodes and an insulator for the electro-static friction stimulus are positioned. The principle of the electrostatic friction stimulus is shown in the bottom-right panel of Figure 2. Without applied voltage, no external force is applied to the contacted skin and the user can perceive a smooth feeling via the flat insulator layer. With applied voltage, the electrodes and the contacted skin are oppositely charged because of the dielectric polarization of the insulator layer. Subsequently, an electrostatic force is applied to the skin, which is attracted toward the electrodes. The electrostatic force can be expressed as follows [32]:

(1) $$F^{\prime }=\mu \left(F+N\right)=\mu (\frac{A\varepsilon \varepsilon_{0}}{2}{\left(\frac{V^{\prime }\left(t\right)}{d}\right)}^{2}+N),$$

where ***F’*** is the resulting frictional force, ***µ*** is the frictional coefficient, ***F*** is the electrostatic force on the fingerpad, ***N*** is the normal force toward the surface, ***ε*** is the relative permeability of the stratum corneum, ***ε***_0_ is the vacuum permeability, ***A*** is the overlap area between the fingerpad and the electrode, ***V′***(***t***) is the applied voltage across the stratum corneum, and ***d*** is the thickness of the stratum corneum.

**Figure 3:**
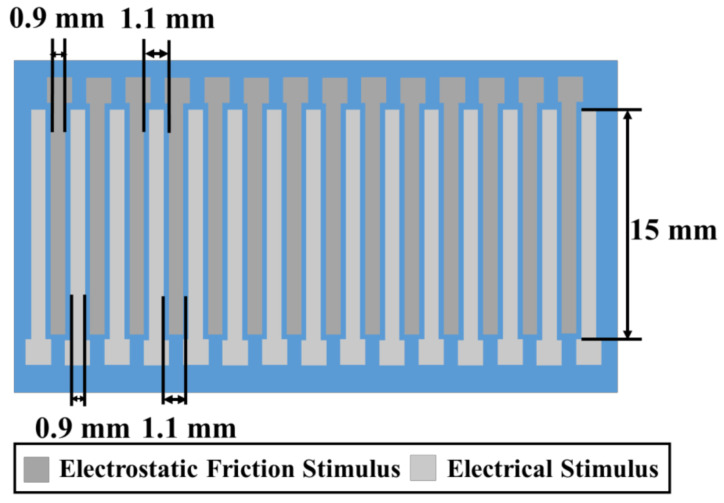
Electrode design of the tactile display.

The electrostatic force increases the resulting frictional force when a user slides his/her fingerpad on the insulator layer. In this study, the width and length of the electrodes used for the electrostatic friction stimulus were 0.9 and 15 mm, respectively, as shown in Figure 3. The thickness of the electrodes was 100 nm. To arrange the electrodes for the electrical stimulus between the electrodes for the electrostatic friction stimulus, the separation between electrodes for the electrostatic friction stimulus is designed to be 1.1 mm. The electrodes were made of chromium, whereas the insulator layer was made of SiO_2_. The insulator layer was 4-μm thick.

The electrodes used to provide an electrical stimulus to the user are positioned on the insulator layer, which is used to induce the electrostatic friction stimulus. The principle of the electrical stimulus is shown in the bottom-left panel of Figure 2. As discussed above, the electrical stimulus requires at least two electrodes, one of which must be connected to a high-voltage terminal, while the other must be grounded. When a user touches the two electrodes, an electrical current passes through them owing to their mutual potential difference, consequently stimulating tactile mechanoreceptors inside the skin. Merkel cells, or Meissner corpuscles, which are located near the surface of the fingerpad, are stimulated by the electrical stimulus, allowing the user to sense a vibration or pressure, depending on the waveform and frequency of the applied current. In this study, the width and length of the electrodes for the electrical stimulus were 0.9 and 15 mm, as shown in Figure 3. The thickness of the electrodes was 100 nm. The separation between the electrodes for the electrostatic friction stimulus was designed to be 1.1 mm. The electrodes were formed with chromium.

The proposed tactile display permits users to perceive both electrical and electrostatic friction stimuli simultaneously by sliding their fingerpad along the surface of the display. By controlling the electric current for the electrical stimulus and the amount of voltage transmitted to the electrodes for the electrostatic friction stimulus, the senses conveyed by these tactile stimuli can be independently controlled.

## 3. Fabrication Process

**Figure 4:**
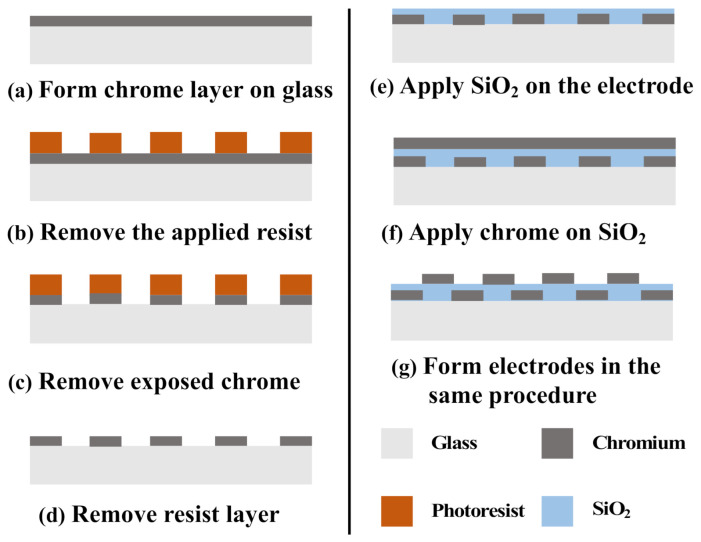
Fabrication process of the proposed tactile display.

**Figure 5:**
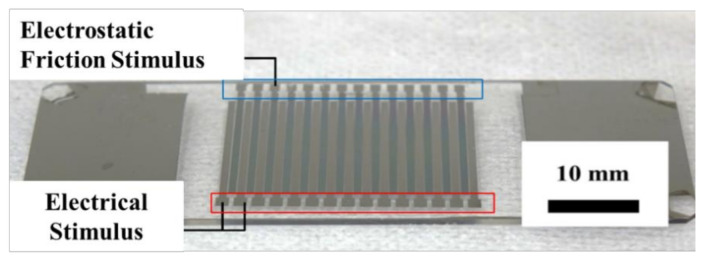
Depiction of the fabricated tactile display.

Figure 4 demonstrates the fabrication process of the proposed tactile display. First, a glass substrate was submerged in a piranha solution to remove organic matter. Chromium was subsequently deposited onto the glass plate for 7 min through sputtering, as shown in Figure 4a. To generate an electrode pattern for the electrostatic friction stimulus, a 2.5-μm-thick positive photoresist layer was formed on the chromium layer through spin coating. The photoresist layer was then exposed to UV light with a photomask, and the exposed area was selectively dissolved with a photoresist developer solution, as shown in Figure 4b. After dissolving the exposed area of the photoresist layer, the bare chromium layer was dissolved with a chromium etching solution, and the electrode pattern for the electrostatic friction stimulus was obtained, as shown in Figure 4c,d. SiO_2_ was deposited on the electrode pattern to form an insulator layer through sputtering for 400 min, as shown in Figure 4e. A portion of the electrode pattern was covered with Kapton tape to form the connective component between the electrodes and a high-voltage power supply. To form an electrode pattern for the electrical stimulus, a chromium layer was deposited on a SiO_2_ layer through a 7-min sputtering process, as shown in Figure 4f. Chromium etching with photoresist patterning was then used to finalize this electrode pattern, as shown in Figure 4g. Figure 5 depicts the fabricated tactile display.

## 4. Experimental Procedure

In order to evaluate the tactile display, we conducted three experiments. In the first and second experiments, the characteristics of each stimulus were evaluated. In the last experiment, multiple stimuli were evaluated. Eight male participants (average age: 23.25 years, SD: 1.01) participated in the experiments. 

### 4.1. Experimental Setup

**Figure 6:**
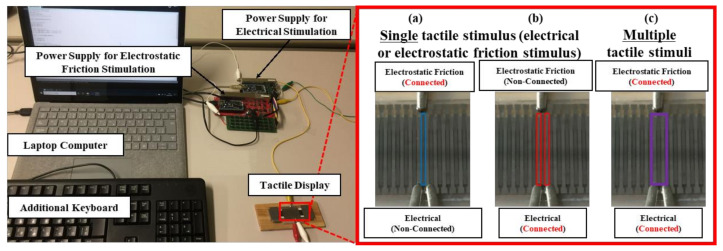
Photograph of the experimental setup.

Figure 6 illustrates the experimental setup, which consisted of a laptop computer (Surface Laptop, Microsoft Corp., Redmond, WA, USA), microcontrollers (mbed LPC 1768, ARM Ltd., Cambridge, UK), a high-current supply for the electrical stimulus (MHV 12-300S10P, Bellnix Co., Ltd., Saitama, Japan), a high-voltage power supply for the electrostatic friction stimulus (MHV 12-1.0k2000P, Bellnix Co., Ltd., Saitama, Japan), an additional keyboard (KB212-B, Dell Inc., Round Rock, TX, USA), and the fabricated tactile display. The values of the voltage and current were controlled by pressing the assigned buttons on the keyboard. According to the input, signals were sent to the current and voltage power supplies via the microcontroller. The connections for each stimulus condition are shown in the right panel of Figure 6. The voltage waveform for the electrostatic friction stimulus was square and the duty cycle of the voltage was 20% because the power supply can only generate positive voltage. The current waveform was a pulse, and the pulse width of the current was fixed at 200 µs [33,34]. As an electrical stimulus can occasionally cause pain depending on its waveform condition, we selected the pulse width used in related studies to provide painless stimulation. Before the experiments, we instructed the participants to slide their dominant fingerpad at a speed of 50 mm/s using a marker shown on a smartphone.

### 4.2. Evaluation of Electrical Stimulus

Electrical stimulation can be perceived as pressure or vibration according to the stimulation condition. As the concept underlying the proposed tactile display is to combine roughness and friction, we first characterized how the electrical stimulus was perceived according to its stimulus condition. In the experiments, the participants were seated in a chair and instructed to slide their index finger across the display to determine the threshold current of the electrical stimulus under each frequency by adjusting the intensity of the current. As the threshold current is often affected by individual skin characteristics, each subject had to be subjectively evaluated to determine the threshold current. Once this had been accomplished, we provided the electrical stimulus, whose peak current was 1.3, 1.6, or 1.9 times higher than the threshold current, to the participants under each frequency. The participants were instructed to slide their index finger across the tactile display at a speed of 50 mm/s and were then asked how they perceived the electrical stimulus using a seven-point Likert scale (vibration to pressure). The frequency was set at 20 Hz, 40 Hz, 80 Hz, 160 Hz, or 320 Hz in order to evaluate a wide frequency range. Each condition was tested once. A total of 15 trials were conducted for each participant.

### 4.3. Evaluation of Electrostatic Friction Stimulus

An electrostatic friction stimulus physically provides friction. As high voltage can cause the breakdown of an electrode insulator [35], the amount of electric current passing through the insulator must be decreased. To achieve this, the duty cycle of the applied voltage is decreased. However, this action can cause a periodic change in the provided friction. In this experiment, we investigated how participants perceived periodic friction. The participants were instructed to slide their index finger across the tactile display, and the threshold voltage was determined under each frequency by adjusting the intensity of the applied voltage. Like an electrical stimulus, an electrostatic friction stimulus can also be affected by individual skin characteristics and was therefore determined by subjective experimentation. We provided the electrostatic friction stimulus, whose peak voltage was 1.3, 1.6, or 1.9 times higher than the threshold voltage, under each frequency. The participants slid their index finger across the tactile display at a speed of 50 mm/s and were then asked how they perceived the electrostatic friction stimulus via a seven-point Likert scale (discontinuous friction to continuous friction). The frequency was set at 20 Hz, 40 Hz, 80 Hz, 160 Hz, or 320 Hz in order to evaluate a wide frequency range. The threshold voltages and currents obtained in previous evaluations were applied in this evaluation as well. Each condition was tested once. A total of 15 trials were conducted for each participant.

### 4.4. Evaluation of Multiple Stimuli

**Figure 7:**
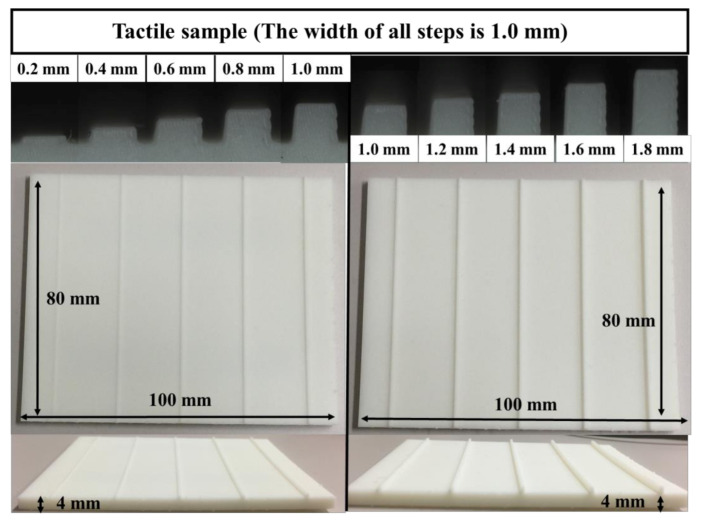
Photograph of the tactile samples.

It is known that an electrical stimulus can reproduce bar shapes. We therefore considered whether multiple stimuli using electrical stimulation could reproduce even more realistic bar shapes. In this experiment, we evaluated how the multiple stimuli condition affected perceived senses. We asked the participants to slide their finger across the tactile display at a speed of 50 mm/s under multiple stimuli. The participants then compared the stimuli with 3D-printed bumps, as shown in Figure 7, and selected the bars that provided a stimulus that was the most similar to the multiple stimuli. The heights of the bars were 0.2 mm, 0.4 mm, 0.6 mm, 0.8 mm, 1.0 mm, 1.2 mm, 1.4 mm, 1.6 mm, and 1.8 mm. The width of all bumps was determined by the electrode and fixed at 1 mm. Additionally, the participants rated the similarity between the multiple stimuli and the bars (1 to 7). The frequencies of the electrical stimulus and the electrostatic stimulus were fixed at 320 Hz, according to the results of the previous experiments. In this evaluation, we widened the range of the peak value to evaluate various conditions, and the peak voltage and peak current were set to 0.0, 1.0, 1.3, 1.6, and 1.9 times higher than the threshold values. The threshold values were obtained from the previous experiments. A total of 24 trials were performed on each participant depending on the combination of voltage and current without the stimulus condition.

## 5. Results and Discussion

### 5.1. Evaluation of the Electrical Stimulus

**Figure 8:**
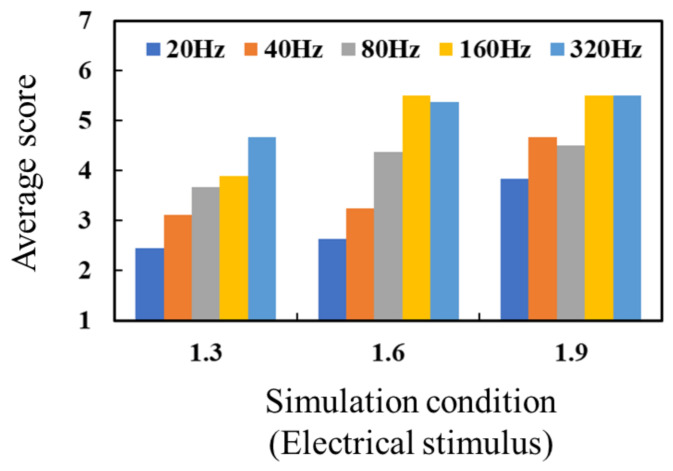
Average score of perceived pressure via the electrical stimulus, with 1 meaning vibration and 7 meaning pressure.

**Figure A1:**
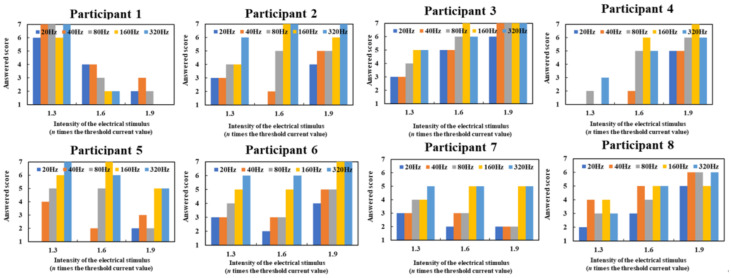
Score of electrical stimulus for each subject.

Figure 8 shows the experimental results for the electrical stimulus, with more detailed data provided in Figure A1 in Appendix A. The average values of the threshold current were 3.11 mA (SD: 0.69 mA), 2.81 mA (SD: 0.78 mA), 2.63 mA (SD: 0.77 mA), 2.16 mA (SD: 0.791 mA), and 2.30 mA (SD: 0.80 mA) under the frequencies of 20 Hz, 40 Hz, 80 Hz, 160 Hz, and 320 Hz. A higher score meant that the participant tended to perceive pressure. The intensity of the electrical stimulus did not greatly affect the score. The participants tended to perceive the electrical stimulus as a vibration under the low-frequency condition. As the frequency of the electrical stimulus was increased, the participants tended to perceive the electrical stimulus as pressure. The perceived pressure score appeared to be saturated under the electrical stimulus at a frequency of 160 Hz and 320 Hz, which indicates that this electrical stimulus was sufficient to provide pressure to users. In Ara’s study [34], an electrical stimulus at a frequency of approximately 50 Hz or less provided a tactile sense, such as “fluttering” or “vibrating,” to the participants. The application of an electrical stimulus at a frequency of 150 Hz or higher allowed the participants to perceive a tactile sense such as “pin-pricking.” This experimental result aligned with that of Ara and supported the electrical stimulus theory proposed in [14,15]. We considered that the proposed tactile display has the same capability to provide electrical stimulation as those described in related studies, although the shape and resolution of the electrodes in the displays were different. The electrical current for the electrical stimulus flows between two electrodes in contact with the skin. The electrical current subsequently flows through the shallow part of the skin because the path of the electrical current in this part has a lower impedance than that in deeper parts of the skin. Thus, it is thought that electrical stimulation stimulates tactile mechanoreceptors, such as Merkel cells and Meissner corpuscles. Muniak et al. revealed the frequency responses of these tactile mechanoreceptors to a vibration stimulus [36]. According to their results, Merkel cells respond to vibration at a frequency of 20 Hz to 100 Hz, while Meissner corpuscles respond to vibration at a frequency of 20 Hz to 300 Hz. Thus, it is possible that as these mechanoreceptors respond to an electrical stimulus at a lower frequency, humans are able to perceive vibration via this low-frequency stimulus. Conversely, however, humans cannot perceive vibration at a higher-frequency electrical stimulus, even though Meissner corpuscles are capable of responding to vibration at a higher frequency. To explain this, detailed investigations into the relationship between an electrical stimulus and mechanoreceptor responses are needed in the future. For the purposes of the present study, the finding that an electrical stimulus at a frequency of 320 Hz can provide the tactile sense of pressure was considered sufficient.

### 5.2. Evaluation of Electrostatic Friction Stimulus

**Figure 9:**
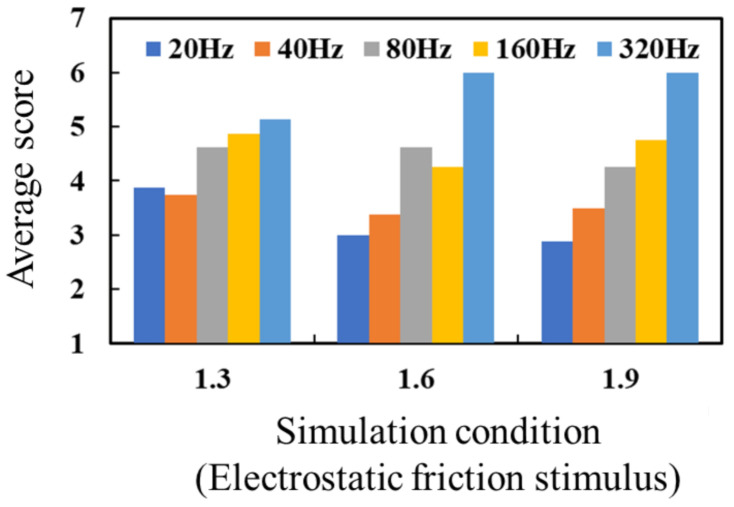
Average score of perceived friction via the electrostatic friction stimulus, with 1 meaning discontinuous friction and 7 meaning continuous friction.

**Figure A2:**
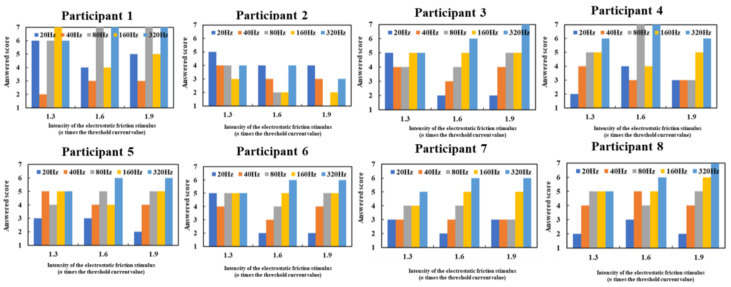
Score of electrostatic friction stimulus for each subject.

Figure 9 shows the experimental results for the electrostatic friction stimulus. The data for each subject are shown in Figure A2. The average values of the threshold voltage were 77.02 V (SD: 51.33 V), 66.66 V (SD: 46.78 V), 37.12 V (SD: 27.11 V), 29.36 V (SD: 21.87 V), and 28.71 V (SD: 21.97 V) under the frequencies of 20 Hz, 40 Hz, 80 Hz, 160 Hz, and 320 Hz. A higher score meant that the participant tended to perceive continuous friction. The score for perceived continuous friction increased as the frequency of the electrostatic friction stimulus was increased, regardless of the intensity of the stimulus. According to Equation (1), the electrostatic force, which changes the frictional force between the fingerpad and the electrode, should be periodically changed according to the frequency of the applied voltage. Thus, an electrostatic force stimulus with a low frequency induces a slow change in friction, which in turn results in a slow vibration on the contacted skin. This vibration was perceived as a discontinuous stimulus, and the participants accordingly gave it a low score. Actually, in a related study, the participants tended to perceive a bumpy tactile sense under a low-frequency condition [37]. We confirmed that our results for low frequency corresponded to the results of the related study. On the other hand, the electrostatic force stimulus with a high frequency caused a high vibration on the contacted skin, according to the abovementioned theory. This vibration provided a continuous tactile sense such that participants were not able to distinguish the interval of each stimulus. In this experiment, we concluded that the electrostatic friction stimulus with a frequency of 320 Hz was suitable to provide continuous friction and was therefore applied in Section 4.3.

### 5.3. Evaluation of Multiple Stimuli

**Figure 10:**
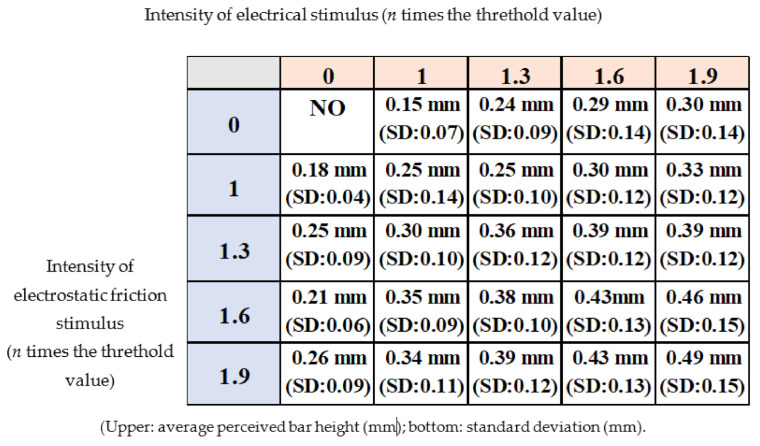
The height of the bar as perceived by the experimental participants.

**Figure 11:**
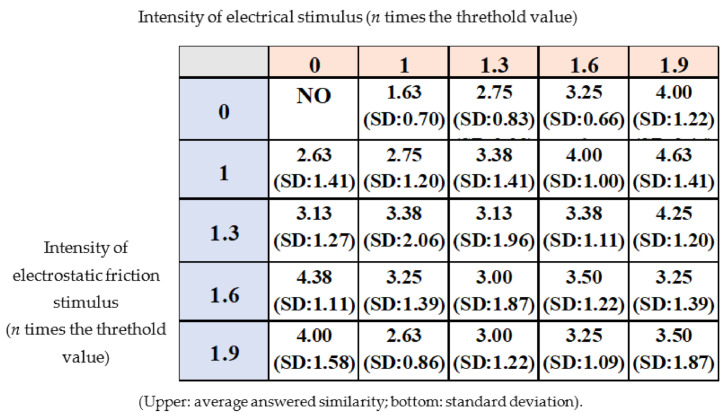
The similarity of the step as rated by the experimental participants.

Figure 10 shows the relationship between the electrical and electrostatic friction stimuli conditions and the average of the height reported by the participants. The average of the answered similarity under each stimulus condition is shown in Figure 11. More detailed data are provided in Table A1 and Table A2 in Appendix B and Appendix C. The average height was 0.30 mm or less when only the electrical stimulus or the electrostatic friction stimulus was provided. In the case of the electrical stimulus, the average height was nearly the same as compared to the results of the related study [15]. The results for the electrostatic friction stimulus revealed that it had the potential to reproduce a bar shape with a height of 0.26 mm or less under this experimental condition. The answered similarity was a maximum of about 4 (60%) for only the electrical stimulus or only the electrostatic friction stimulus.

In the case of the multiple tactile stimuli, the participants tended to perceive a higher height, as shown in the bottom-right of Figure 10. This result indicates that multiple tactile stimuli have the potential to enhance their resulting intensity, although their characteristics are different. The resulting average height seemed to be the same or less than the heights reproduced by the electrical stimulus and the electrostatic friction stimulus. If so, then one stimulus simply enhances another stimulus, and no unique interaction is induced. However, the enhancement of the intensity of the resulting tactile stimulus using two different stimuli is interesting. Actually, it was considered that a single stimulus may reproduce shapes of greater height when a higher peak voltage is applied. Higher voltage and current can increase the power supply but also runs the risk of electric shock and pain. Thus, the result obtained in this regard is important, as it demonstrates that the intensity of the tactile stimulus requires lower voltage or current. The score of perceived similarity was not significantly increased. The multiple stimuli condition (electrical stimulus: 1.9, electrostatic friction stimulus: 1.0) exhibited the highest score, which was nearly the same as the highest score for the single stimulus. Thus, we concluded that multiple stimuli can increase their perceived intensity without increasing their peak value, although they are not effective in enhancing the similarity under the stimulation conditions in this study.

## 6. Conclusions

An electrode-type tactile display combining an electrical stimulus and an electrostatic friction stimulus was evaluated to verify its potential to reproduce a virtual bar shape via multiple tactile stimuli. We characterized each stimulus to determine the conditions in which pressure or continuous friction was provided. Then, we compared the single tactile stimulus or the multiple tactile stimuli with real bar shapes. The results showed that participants tended to perceive the multiple tactile stimuli as a higher bar ranging up to 0.5 mm in height. Multiple stimuli can thus improve the intensity of the tactile stimulus without higher peak voltage or current and are therefore effective in the design of safer systems. In terms of tactile similarity, on the other hand, the maximum perceived rating was almost 4 and the current stimuli condition did not improve tactile similarity as compared with a single stimulus. We believe that the proposed tactile display will contribute to the presentation of shapes via touchscreens. Although conventional electrode-type tactile displays can be embedded in touchscreens, they cannot present shapes of greater height. Our results suggest that the proposed tactile display can expand the shapes presented via touchscreens while retaining affinity to them. Further miniaturization of the tactile display stimulator to present shapes at higher resolutions is possible. In our future work, we plan to optimize the current waveform and the voltage waveform, which are related to the presented tactile sense, to achieve a more realistic tactile sense.

## Appendix B

**Table A1:** Heights reported by each participant.

		Participant A			
	**0**	**1**	**1.3**	**1.6**	**1.9**
**0**	**NO**	**0.10 mm**	**0.10 mm**	**0.20 mm**	**0.20 mm**
**1**	**0.10 mm**	**0.10 mm**	**0.10 mm**	**0.10 mm**	**0.10 mm**
**1.3**	**0.10 mm**	**0.20 mm**	**0.40 mm**	**0.40 mm**	**0.40 mm**
**1.6**	**0.10 mm**	**0.30 mm**	**0.40 mm**	**0.40 mm**	**0.60 mm**
**1.9**	**0.20 mm**	**0.30 mm**	**0.40 mm**	**0.50 mm**	**0.60 mm**
		Participant B			
	**0**	**1**	**1.3**	**1.6**	**1.9**
**0**	**NO**	**0.20 mm**	**0.30 mm**	**0.40 mm**	**0.40 mm**
**1**	**0.20 mm**	**0.30 mm**	**0.40 mm**	**0.50 mm**	**0.50 mm**
**1.3**	**0.30 mm**	**0.40 mm**	**0.40 mm**	**0.50 mm**	**0.50 mm**
**1.6**	**0.30 mm**	**0.50 mm**	**0.50 mm**	**0.60 mm**	**0.60 mm**
**1.9**	**0.40 mm**	**0.50 mm**	**0.60 mm**	**0.60 mm**	**0.70 mm**
		Participant C			
	**0**	**1**	**1.3**	**1.6**	**1.9**
**0**	**NO**	**0.20 mm**	**0.20 mm**	**0.20 mm**	**0.20 mm**
**1**	**0.20 mm**	**0.20 mm**	**0.20 mm**	**0.30 mm**	**0.30 mm**
**1.3**	**0.20 mm**	**0.20 mm**	**0.30 mm**	**0.30 mm**	**0.30 mm**
**1.6**	**0.20 mm**	**0.30 mm**	**0.30 mm**	**0.40 mm**	**0.30 mm**
**1.9**	**0.20 mm**	**0.30 mm**	**0.30 mm**	**0.30 mm**	**0.40 mm**
		Participant D			
	**0**	**1**	**1.3**	**1.6**	**1.9**
**0**	**NO**	**0.20 mm**	**0.20 mm**	**0.20 mm**	**0.20 mm**
**1**	**0.20 mm**	**0.20 mm**	**0.20 mm**	**0.30 mm**	**0.30 mm**
**1.3**	**0.30 mm**	**0.40 mm**	**0.40 mm**	**0.40 mm**	**0.40 mm**
**1.6**	**0.20 mm**	**0.50 mm**	**0.50 mm**	**0.50 mm**	**0.60 mm**
**1.9**	**0.20 mm**	**0.50 mm**	**0.50 mm**	**0.60 mm**	**0.60 mm**
		Participant E			
	**0**	**1**	**1.3**	**1.6**	**1.9**
**0**	**NO**	**0.00 mm**	**0.40 mm**	**0.60 mm**	**0.60 mm**
**1**	**0.20 mm**	**0.60 mm**	**0.40 mm**	**0.40 mm**	**0.40 mm**
**1.3**	**0.40 mm**	**0.40 mm**	**0.60 mm**	**0.60 mm**	**0.60 mm**
**1.6**	**0.20 mm**	**0.40 mm**	**0.40 mm**	**0.60 mm**	**0.60 mm**
**1.9**	**0.40 mm**	**0.20 mm**	**0.40 mm**	**0.40 mm**	**0.60 mm**
		Participant F			
	**0**	**1**	**1.3**	**1.6**	**1.9**
**0**	**NO**	**0.20 mm**	**0.20 mm**	**0.20 mm**	**0.20 mm**
**1**	**0.20 mm**	**0.20 mm**	**0.20 mm**	**0.40 mm**	**0.40 mm**
**1.3**	**0.20 mm**	**0.20 mm**	**0.20 mm**	**0.20 mm**	**0.20 mm**
**1.6**	**0.20 mm**	**0.20 mm**	**0.20 mm**	**0.20 mm**	**0.20 mm**
**1.9**	**0.20 mm**	**0.20 mm**	**0.20 mm**	**0.20 mm**	**0.20 mm**
		Participant G			
	**0**	**1**	**1.3**	**1.6**	**1.9**
**0**	**NO**	**0.20 mm**	**0.30 mm**	**0.30 mm**	**0.40 mm**
**1**	**0.20 mm**	**0.20 mm**	**0.30 mm**	**0.20 mm**	**0.40 mm**
**1.3**	**0.30 mm**	**0.40 mm**	**0.40 mm**	**0.40 mm**	**0.40 mm**
**1.6**	**0.30 mm**	**0.40 mm**	**0.40 mm**	**0.40 mm**	**0.40 mm**
**1.9**	**0.30 mm**	**0.30 mm**	**0.30 mm**	**0.40 mm**	**0.40 mm**
		Participant H			
	**0**	**1**	**1.3**	**1.6**	**1.9**
**0**	**NO**	**0.10 mm**	**0.20 mm**	**0.20 mm**	**0.20 mm**
**1**	**0.10 mm**	**0.20 mm**	**0.20 mm**	**0.20 mm**	**0.20 mm**
**1.3**	**0.20 mm**	**0.20 mm**	**0.20 mm**	**0.30 mm**	**0.30 mm**
**1.6**	**0.20 mm**	**0.30 mm**	**0.30 mm**	**0.30 mm**	**0.40 mm**
**1.9**	**0.20 mm**	**0.40 mm**	**0.40 mm**	**0.40 mm**	**0.40 mm**

## Appendix C

**Table A2:** Perceived similarity reported by each participant.

		Participant A			
	**0**	**1**	**1.3**	**1.6**	**1.9**
**0**	**NO**	**1**	**2**	**2**	**3**
**1**	**1**	**2**	**4**	**4**	**4**
**1.3**	**1**	**2**	**1**	**3**	**4**
**1.6**	**4**	**3**	**1**	**5**	**2**
**1.9**	**3**	**4**	**4**	**4**	**5**
		Participant B			
	**0**	**1**	**1.3**	**1.6**	**1.9**
**0**	**NO**	**2**	**4**	**4**	**5**
**1**	**4**	**2**	**3**	**4**	**6**
**1.3**	**4**	**5**	**5**	**4**	**5**
**1.6**	**5**	**4**	**5**	**2**	**3**
**1.9**	**2**	**2**	**2**	**4**	**1**
		Participant C			
	**0**	**1**	**1.3**	**1.6**	**1.9**
**0**	**NO**	**1**	**2**	**4**	**5**
**1**	**1**	**3**	**4**	**4**	**6**
**1.3**	**3**	**2**	**4**	**4**	**5**
**1.6**	**5**	**1**	**2**	**2**	**3**
**1.9**	**5**	**2**	**3**	**2**	**1**
		Participant D			
	**0**	**1**	**1.3**	**1.6**	**1.9**
**0**	**NO**	**1**	**2**	**3**	**6**
**1**	**4**	**2**	**4**	**4**	**5**
**1.3**	**2**	**4**	**2**	**3**	**6**
**1.6**	**6**	**3**	**1**	**3**	**1**
**1.9**	**6**	**2**	**1**	**2**	**6**
		Participant E			
	**0**	**1**	**1.3**	**1.6**	**1.9**
**0**	**NO**	**1**	**2**	**3**	**3**
**1**	**4**	**4**	**3**	**4**	**5**
**1.3**	**4**	**5**	**3**	**5**	**5**
**1.6**	**2**	**6**	**6**	**5**	**6**
**1.9**	**2**	**3**	**4**	**4**	**3**
		Participant F			
	**0**	**1**	**1.3**	**1.6**	**1.9**
**0**	**NO**	**2**	**3**	**3**	**4**
**1**	**2**	**3**	**2**	**2**	**2**
**1.3**	**4**	**1**	**1**	**1**	**2**
**1.6**	**4**	**3**	**2**	**3**	**4**
**1.9**	**3**	**2**	**3**	**3**	**3**
		Participant G			
	**0**	**1**	**1.3**	**1.6**	**1.9**
**0**	**NO**	**2**	**3**	**3**	**2**
**1**	**1**	**1**	**1**	**4**	**3**
**1.3**	**2**	**1**	**2**	**4**	**4**
**1.6**	**4**	**2**	**2**	**3**	**3**
**1.9**	**5**	**2**	**2**	**2**	**3**
		Participant H			
	**0**	**1**	**1.3**	**1.6**	**1.9**
**0**	**NO**	**3**	**4**	**4**	**4**
**1**	**4**	**5**	**6**	**6**	**6**
**1.3**	**5**	**7**	**7**	**3**	**3**
**1.6**	**5**	**4**	**5**	**5**	**4**
**1.9**	**6**	**4**	**5**	**5**	**6**

## References

[1] Chouvardas V.G. Tactile displays: A short overview and recent developments. Proceedings of the 5th International Conference on Technology and Automation.

[2] Ishizuka H., Miki N. (2015). MEMS-based tactile displays. Displays.

[3] Pacchierotti C., Sinclair S., Solazzi M., Frisoli A., Hayward V., Prattichizzo D. (2017). Wearable haptic systems for the fingertip and the hand: Taxonomy, review, and perspectives. IEEE Trans. Haptics.

[4] Yamamoto A., Nagasawa S., Yamamoto H., Higuchi T. (2006). Electrostatic tactile display with thin film slider and its application to tactile telepresentation systems. IEEE Trans. Vis. Comput. Graph..

[5] Tzemanaki A., Al G.A., Melhuish C., Dogramadzi S. (2018). Design of a wearable fingertip haptic device for remote palpation: Characterisation and interface with a virtual environment. Front. Robot. AI.

[6] Okamura A.M., Dennerlein J.T., Howe R.D. Vibration feedback models for virtual environments. Proceedings of the IEEE International Conference on Robotics and Automation.

[7] Jang S., Kim L.H., Tanner K., Ishii H., Follmer S. Haptic edge display for mobile tactile interaction. Proceedings of the 2016 CHI Conference on Human Factors in Computing Systems.

[8] Lévesque V., Pasquero J., Hayward V., Legault M. (2005). Display of virtual braille dots by lateral skin deformation: Feasibility study. ACM Trans. Appl. Percept..

[9] Zhao F., Fukuyama K., Sawada H. Compact Braille display using SMA wire array. Proceedings of the IEEE International Workshop on Robot and Human Interactive Communication.

[10] Kajimoto H., Jones L.A. (2019). Wearable tactile display based on thermal expansion of nichrome wire. IEEE Trans. Haptics.

[11] Shao Y., Ma S., Yoon S.H., Visell Y., Holbery J. SurfaceFlow: Large area haptic display via compliant liquid dielectric actuators. Proceedings of the IEEE Haptics Symposium, HAPTICS.

[12] Tomita H., Saga S., Takahashi S., Kajimoto H. A proposal and investigation of displaying method by passive touch with electrostatic tactile display. Proceedings of the Eurohaptics.

[13] Sonar H.A., Gerratt A.P., Lacour S.P., Paik J. (2020). Closed-loop haptic feedback control using a self-sensing soft pneumatic actuator skin. Soft Robot..

[14] Kajimoto H., Kawakami N., Tachi S. Electro-Tactile Display with Tactile Primary Color Approach. Proceedings of the 2004 IEEE/RSJ International Conference on Intelligent Robots and Systems.

[15] Yem V., Kajimoto H. (2016). Comparative evaluation of tactile sensation by electrical and mechanical stimulation. IEEE Trans. Haptics.

[16] Kajimoto H. (2012). Electrotactile display with real-time impedance feedback using pulse width modulation. IEEE Trans. Haptics.

[17] Biet M., Giraud F., Lemaire-Semail B. (2007). Squeeze film effect for the design of an ultrasonic tactile plate. IEEE Trans. Ultrason. Ferroelectr. Freq. Control.

[18] Mallinckrodt E., Hughes A.L., Sleator W. (1953). Perception by the skin of electrically induced vibrations. Science.

[19] Strong R.M., Troxel D.E. (1970). An electrotactile display. IEEE Trans. Man Mach. Syst..

[20] Bergmann Tiest W.M. (2010). Tactual perception of material properties. Vis. Res..

[21] Okamoto S., Nagano H., Yamada Y. (2013). Psychophysical dimensions of tactile perception of textures. IEEE Trans. Haptics.

[22] Ballesteros S., Reales J.M., De Leon L.P., Garcia B. The perception of ecological textures by touch: Does the perceptual space change under bimodal visual and haptic exploration?. Proceedings of the World Haptics Conference.

[23] Shirado H., Maeno T. Modeling of human texture perception for tactile displays and sensors. Proceedings of the World Haptics Conference.

[24] Guest S., Dessirier J.M., Mehrabyan A., McGlone F., Essick G., Gescheider G., Fontana A., Xiong R., Ackerley R., Blot K. (2011). The development and validation of sensory and emotional scales of touch perception. Atten. Percept. Psychophys..

[25] Yoshioka T., Bensmaïa S.J., Craig J.C., Hsiao S.S. (2007). Texture perception through direct and indirect touch: An analysis of perceptual space for tactile textures in two modes of exploration. Somatosens. Mot. Res..

[26] Hollins M., Risner S.R. (2000). Evidence for the duplex theory of tactile texture perception. Percept. Psychophys..

[27] Ito K., Okamoto S., Yamada Y. (2019). Tactile texture display with vibrotactile and electrostatic friction stimuli mixed at appropriate ratio presents better. ACM Trans. Appl. Percept..

[28] Komurasaki S., Kajimoto H., Ishizuka H. (2019). Fundamental perceptual characterization of an integrated tactile display with electrovibration and electrical stimuli. Micromachines.

[29] Kajimoto H., Kawakami N., Tachi S., Inami M. (2004). SmartTouch: Electric skin to touch the untouchable. IEEE Comput. Graph. Appl..

[30] Haghighi Osgouei R., Kim J.R., Choi S. (2020). Data-driven texture modeling and rendering on electrovibration display. IEEE Trans. Haptics.

[31] Jiao J., Member S., Zhang Y., Member S., Wang D., Member S., Visell Y., Cao D., Guo X., Sun X. Data-driven rendering of fabric textures on electrostatic tactile displays. Proceedings of the 2018 IEEE Haptics Symposium (HAPTICS).

[32] Vardar Y., Guclu B., Basdogan C. (2017). Effect of waveform on tactile perception by electrovibration displayed on touch screens. IEEE Trans. Haptics.

[33] Kaczmarek K.A., Webster J.G., Bach-y-Rita P., Tompkins W.J. (1991). Electrotactile and vibrotactile displays for sensory substitution systems. IEEE Trans. Biomed. Eng..

[34] Ara J., Hwang S.H., Song T., Khang G. (2014). Effects of the stimulus parameters on the tactile sensations elicited by single-channel transcutaneous electrical stimulation. Int. J. Precis. Eng. Manuf..

[35] Ishizuka H., Suzuki K., Terao K., Takao H., Shimokawa F., Kajimoto H. Development of a multi-electrode electrovibration tactile display with 1 mm resolution. Proceedings of the 2017 IEEE World Haptics Conference.

[36] Muniak M.A., Ray S., Hsiao S.S., Dammann J.F., Bensmaia S.J. (2007). The neural coding of stimulus intensity: Linking the population response of mechanoreceptive afferents with psychophysical behavior. J. Neurosci..

[37] Bau O., Poupyrev I., Israr A., Harrison C. TeslaTouch: Electrovibration for touch surfaces. Proceedings of the ACM Symposium on User Interface Software and Technology.

